# Assessment of Radon Levels in Drinking Water Wells in St. Catherine, Jamaica

**DOI:** 10.5696/2156-9614-7.16.31

**Published:** 2017-12-18

**Authors:** Leonard Smith, Mitko Voutchkov

**Affiliations:** Department of Physics, Faculty of Science and Technology, The University of the West Indies at Mona, Kingston, Jamaica

**Keywords:** Radon, radiation, cancer, drinking water, wells, environmental health, public health, Jamaica, St. Catherine, water quality

## Abstract

**Background.:**

Radon is a known carcinogen and contaminant in drinking water wells, but is not monitored in drinking water quality programs in Jamaica.

**Objective.:**

The present study was conducted to obtain radon data in local drinking water and evaluate potential health risks. The data will contribute to determining the level of compliance to public health criteria for radon and to develop a monitoring program based on the identified risks.

**Methods.:**

This study assesses the concentration of radon in 22 drinking water wells in the parish of St. Catherine, Jamaica. Samples were collected for radon, with 12 other measurements gathered including pH, conductivity, TDS, alkalinity, hardness, phosphates, nitrates, chloride, sulfates, turbidity, well depth and geological features. The data were analyzed for compliance to international limits and association with geological and other parameters.

**Results.:**

The average radon level was 18 Bq/L ± 2 Bq/L and varied from a low of 11 Bq/L ± 1 Bq/L to a high of 41 Bq/L ± 1 Bq/L. There was a positive correlation between radon levels and both alkalinity and turbidity. No relationship of any significance, however, was identified with the other physicochemical parameters. All the study results fell within the European Union (EU) limit of 100 Bq/L, and well within the United States Environmental Protection Agency (USEPA) limit of 147 Bq/L. Most of the wells in this parish have radon levels exceeding the proposed USEPA limit of 11 Bq/L. The proposed limits are intended to support radon mitigation programs to manage radon in air. No limits are provided in the newest edition of the World Health Organization's (WHO) Guidelines for Safe Drinking Water Quality.

**Conclusions.:**

Most wells in the study area met existing international limits. Almost all, however, did not meet the proposed USEPA limit for locations without radon mitigation programs. This indicates the need to establish national screening levels for radon, consistent with WHO and USEPA recommendations.

## Introduction

Radon is a highly radioactive gaseous element. It is released from the alkaline earth metal radium which is also a bi-product of the decay of uranium. Uranium is found in most rocks naturally. Due to its radioactivity, natural occurrence and risk to health, radon has been the subject of many studies.^1^ It has been mainly studied locally in soil, however, it has not been sufficiently studied in drinking water.

Numerous studies have confirmed the strong correlation of exposure to radon with lung cancer.^2–5^ Al-Zoughool and others conducted a review of the literature on the health risks associated with radon and confirmed that it is an established human lung carcinogen.^1^ This was based on epidemiological data and supported by experimental evidence in cell cultures and laboratory animals. They found that radon is suspected to be the next highest cause of cancer deaths after cigarette smoking among miners.^1^ The present study focuses on radon in water. Radon in water can be ingested, which can lead to stomach and bladder cancers, but the evidence on this relationship is unclear. When water is aerosolized, for example in bathroom showers, it is inhaled, and studies indicate that this is an important form of radon exposure from water.^4^

The present study focused on the occurrence of radon in wells in the parish of St. Catherine, Jamaica. St. Catherine has a population of 518,345, the second largest in Jamaica, and the single largest community, Portmore, reported a population of 182,000 in the 2012 census. This parish has around 42 wells, the largest concentration of well sources locally. This comprises 98% of all sources and provides water to most of the parish population. Small community catchment tanks and river water sources are the only other types of water supply. Most of these wells and other water sources are government owned. Wells have been determined to be a more likely source of radon than other water sources due to their depth and connection to groundwater.^4^ This combination of factors adds to the importance of the present study.

This study will contribute to data needed to assess the relative risks associated with radon for national public health programs, as a risk-based approach to public health intervention is a best practice. It will help local health professionals to understand the relative risks associated with environmental contaminants and to be more effective in their allocation of resources. This will ultimately assist Jamaica in meeting its commitments to radiation safety requirements as a member country of the International Atomic Energy Agency (IAEA).

## Methods

The present study employed a cross-sectional study design. Samples were collected from wells over a period of 4 weeks. The target sample size was the entire population of 42 wells as provided by the local water provider. A few samples were also taken from drinking water taps that were close to wells. The samples were collected from different geological formations comprising two terrain types, alluvium and white limestone. Samples were collected using approved protocols described in ‘Standard Methods for the Analysis of Water and Wastewater’, 21st edition.^6^

**Figure 1 —:**
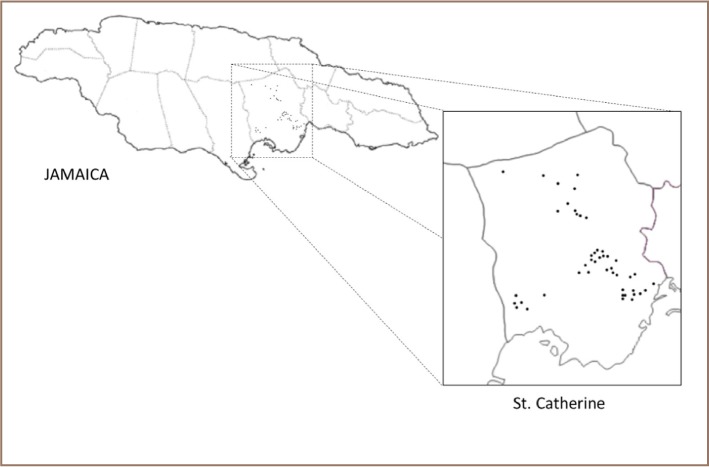
Drinking water well sources in St. Catherine

**Figure 2 —:**
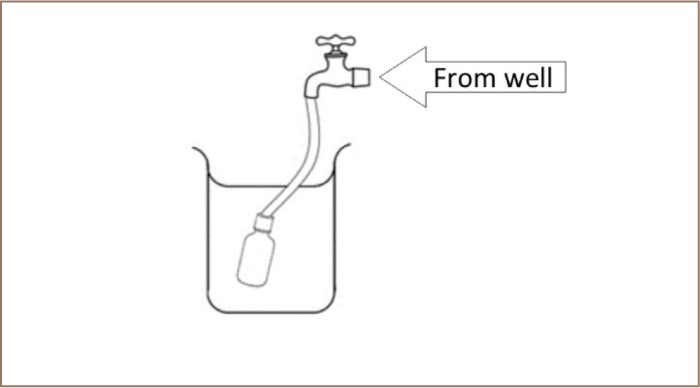
Sample collection method

Over the period of a month, from November 5–26, 2014, samples were collected from each operational well within the parish (*Figure 1*). Samples were collected in the daytime, with sampling times from mid-morning to late afternoon. All the actively operating wells in the parish were targeted in the exercise and pH measurements were taken from the water at each sample site. Sample collection techniques were followed as outlined in the ‘Standard Methods for the Examination of Water and Wastewater’, 21st edition.^6^ Three sample containers were used per location. Two 250 mL amber glass containers with septum caps were used for the sample radon to assess radon (one as duplicate), and a 500 mL polyethylene container for additional parameters as follows: conductivity, total dissolved solids, alkalinity, hardness, phosphates, nitrates, chlorides, sulfates, turbidity and salinity. Samples were collected unfiltered. Preparation of all glassware used during this sampling exercise included washing with phosphate free detergent and rinsing with tap water. Glassware was kept in 1:1 hydrochloric acid and adequately rinsed with deionized water prior to use. Before collecting the sample, the well water tap was allowed to run for 2–3 min or sufficient time for the sample to become representative of the water source. The radon sample was collected by placing the 250 mL container in a 600 mL beaker and filling via a delivery tube placed two-thirds inside. The bottle was filled, displacing the 250 mL volume two or more times. It was allowed to be submerged by the water in the 600 mL beaker and was carefully capped beneath the water surface (*Figure 2*).

**Abbreviations**
*USEPA*: United States Environmental Protection Agency
*WHO*: World Health Organization

Two additional samples were taken from water taps following Standard Methods.^6^ Quality control procedures included the use of a factory calibrated instrument and the analysis of a blank sample to determine background measurements.

The instrument employed in the present study was the Durridge Company Inc's RAD 7 with the RAD H2O accessory, which is reported by the manufacturer to be as accurate as the liquid scintillation method described in the standard methods, while achieving a faster initial reading.^7^ Each result is automatically generated after a 20-minute wait time once the sample is connected to the instrument. The RAD 7 principle is based on a solid state alpha detector. A solid state detector is a semiconductor material (usually silicon) that converts alpha radiation directly to an electrical signal. The RAD 7 instrument was combined with the RAD H_2_O accessory to detect radon in water. This accessory facilitates a closed loop that records the alpha emissions while the sample is agitated with dry air. Although the instrument was factory calibrated, it was “zeroed” by use of a deionized water sample with radon levels greatly reduced by agitation or by allowing to stand until almost all the radon was released. Duplicate measurements were made. The RAD 7 technology has been employed in many studies on radon.^8–10^ The 2-sigma error for the RAD H_2_O (250 mL sample, 20-minute count) at 11 Bq/L is ± 1 Bq/L, which is the same as the liquid scintillation and Lucas cell methods.

The samples were collected in collaboration with the St. Catherine Health Department, and with the assistance of a public health inspector, who routinely collect water samples to assess drinking water quality. Permission for analysis of samples and access to the data was sought from the National Public Health Laboratory who supported the study by providing laboratory reagents and supplies as well as bench space for conducting all testing except radon, and the Ministry of Health's Standards and Regulatory Affairs Division as well as the St. Catherine Health Department for the collection and testing of water samples. Data concerning georeferenced well locations and well depths were received from the National Water Commission, which is the main drinking water provider in this area.

Data analysis methods included measures of central tendency such as mean, measures of distribution on contaminants such as standard deviation and method of comparison of means such as the Students t-test. Pearson's correlation and test of significance were also used. The latter was used to compare the levels of radon which occur in different types of geological formations, well depth, as well as different water quality chemical parameters. Ten additional parameters were measured for all or some of the points, including pH, conductivity, total dissolved solids, alkalinity, hardness, phosphates, nitrates, chloride, sulfates, turbidity and salinity.

## Results

**Figure 3 —:**
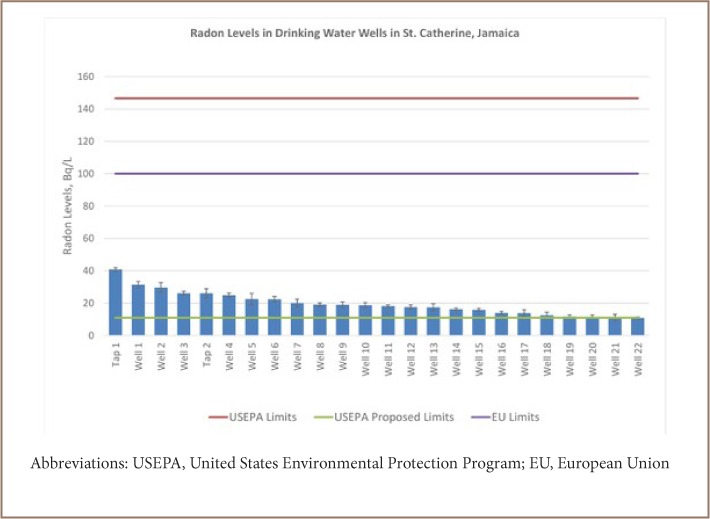
Radon levels in drinking water wells in St. Catherine, Jamaica

The results of radon concentration are shown in Figure 3. They represent samples taken from 22 of the 42 listed wells in the parish of St. Catherine, Jamaica. Results from two drinking water taps are also included. This represents almost all of the active wells at the time. Up to 17 of these wells were no longer being used or were not functional at the time of this study, and the remainder were inaccessible. Radon concentrations ranged from 11 Bq/L ± 1 Bq/L to 41 Bq/L ± 1 Bq/L with an average level of 18 Bq/L ± 2 Bq/L. Levels greater than 20 Bq/L were observed for 9 samples, and 7 had levels below 15 Bq/L. The remaining 8 samples had levels between this range. The highest level was recorded at a tap at a nursery located near the Marlie Mount well.

Radon concentrations were correlated with 10 chemical and physical parameters and the Pearson correlation coefficients are shown in Table 1. Radon showed high correlations with alkalinity, turbidity, hardness and chloride and two of these were statistically significant, shown in bold in the table. Alkalinity, and turbidity showed correlations which were found to be significant. No correlation was found with the remaining parameters, including well depth.

**Table 1 —:**
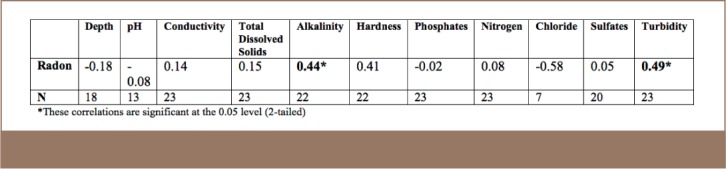
Pearson correlation coefficients between radon concentration in well samples and other water quality parameters

**Table 2 —:**
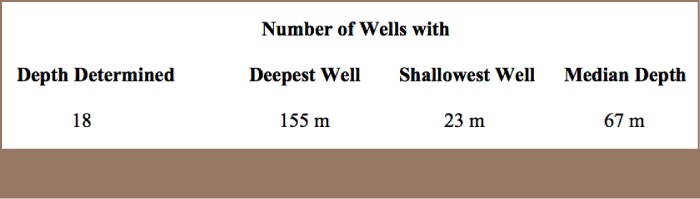
Summary of Well Depth Data

Radon levels in the parish were also correlated with geological formations in the study area. Table 3 provides summary statistics of radon levels observed in alluvium and white limestone geology.

**Table 3 —:**
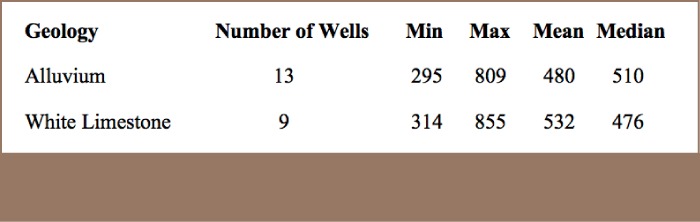
Radon Levels Observed in Alluvium and White Limestone Geology Groups in St. Catherine, Jamaica

No significant differences in radon levels were found by geological group. A comparison of the radon levels in wells in St. Catherine, Jamaica and other countries show that levels in the present study were low compared to some European countries.

## Discussion

Only 22 of the 42 targeted wells were sampled, as most of the wells were not active at the time of sampling. The parish public health inspector responsible for water quality estimated that three additional wells could be sampled, however, these assessments were not performed due to difficulty in accessing these locations. Well inactivity was due to well collapse or malfunctioning pumps, and in a few cases, saline intrusion was reported to be a factor.

The results indicated that most radon levels were satisfactory compared to the European Union (EU) and United States Environmental Protection Agency (USEPA) limit of 147 Bq/L for locations with a radon multimedia mitigation program (MMM). The World Health Organization (WHO) set the guidance level at 100 Bq/L in the third edition of the WHO drinking water guidelines.^18^ However, in the current publication, there are no recommended limits for water. WHO guidelines instead recommend measuring radon in air and provide a limit of 100 Bq/m^3^. Compared to the proposed USEPA limit of 11 Bq/L for locations without an MMM, all except for one sample exceeded this limit. It should be noted that these proposed limits are not directly health derived but are “…intended to promote a more cost-effective multimedia approach to reduce radon risks…”, in other words, to promote MMMs.^19^ This would explain the large disparity between the values, as the 11 Bq/L in water is intended to reduce exposure from radon escape into the air, where it presents a greater threat.^19^ There is no national reference level for air in Jamaica due to the absence of “enhanced state programs to address the health risks from radon in indoor air”, according to the USEPA.^20^ Many communities in Jamaica use water storage tanks. This allows the water to stand for days giving time for radon to escape, reducing or removing it before it is consumed or enters the home. Radon can be removed from water by allowing it to stand for a time or by agitation in air. The WHO recommends that countries establish screening levels for radon in water on the “basis of the national reference level for radon in air and the distribution of radon in the national housing stock”.^18^ The finding of high levels of radon at a children's nursery is one indication of the need to establish a screening program in Jamaica.^20^ The exceedance of the USEPA proposed limit does not indicate a health risk associated with drinking water, however, it supports the need for screening. Since radon is already an established lung carcinogen, such efforts are justified. In addition, further studies on radon are needed locally. Radon levels in this study compare favorably to many European countries, where some levels, as indicated in Table 4, far exceed those presented here.

**Table 4 —:**
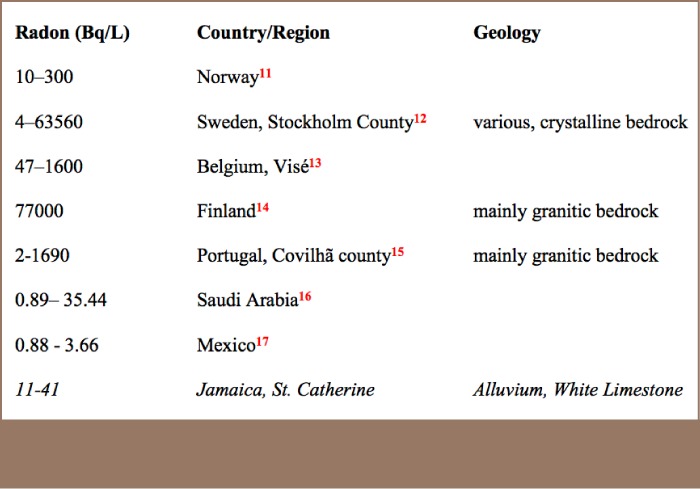
Comparison of radon levels from wells in St. Catherine, Jamaica to wells in other countries.

For the ten chemical and physical parameters assessed, conductivity, TDS, hardness, phosphates, nitrates, and sulfates showed no correlation in the present study. Well depth was also measured and showed no correlation to radon levels. Erdogan et al. found a slight positive correlation in their study of wells in Turkey.^21^ Alkalinity and turbidity, however, showed a clearer trend and positive correlation. Other studies with physico-chemical parameters did not indicate any correlations.^22–24^ The correlation in the present study may not be attributable to radon in the water, but to the radium attached to the soil particulates which contribute to turbidity, in addition to radon already in the water. The data set was insufficient to draw a real conclusion from the correlation assessment for chlorides and pH. There is a confirmed association of turbidity with sediments in water and turbidity was used successfully as a surrogate.^24^ Alkalinity is a measure of the ability of water to neutralize acid. Alkalinity is largely due to carbonates species, but may include other bases as well such as phosphates, silicates and hydroxides. The association of uranium with carbonate rocks is less common than other rocks such as clastic sedimentary rocks, metamorphosed clastic sedimentary rocks, and igneous rocks. Various limestone formations have some amount of epigenetically derived uranium. Several studies associate radon with bauxite, which is a prominent ore yielding aluminum in Jamaica.^25^ St. Catherine did not have good representation of this ore in the studied areas. While this study did not identify a correlation for radon with geological features, other studies have identified correlations with other terrain types such as granite,^23^,^26^,^27^ and pink gray granite versus gray granite.^28^ The present study was performed during the dry season and well water levels are usually lower, and there is a possibility of different results during the wet season.

## Conclusions

The results of the present study indicate that radon levels in drinking wells in St. Catherine are well within the limits set by the USEPA and the EU. Almost all of the results, however, exceeded the USEPA limit proposed for territories without radon mitigation programs. It is hoped that the results of the present study will contribute to the development of water quality programs in Jamaica. Further studies are needed in the remaining parishes in Jamaica, including sampling during both the wet and dry seasons.
